# Factors Contributing to Discriminatory Nursing Care: A Qualitative Study From Nurses' Experiences

**DOI:** 10.1002/nop2.70890

**Published:** 2026-10-04

**Authors:** Aram Karimian, Afsaneh Sadooghiasl, Mitra Khoobi, Eesa Mohammadi, Anoshirvan Kazemnejad

**Affiliations:** ^1^ Department of Nursing, Faculty of Medical Sciences Tarbiat Modares University Tehran Iran; ^2^ Department of Biostatistics, Faculty of Medical Sciences Tarbiat Modares University Tehran Iran

**Keywords:** content analysis, discrimination, moral distress, nursing care, organizational discrimination, qualitative study

## Abstract

**Background:**

Justice and non‐discrimination are core principles of professional nursing ethics. Yet, growing evidence shows that discriminatory behaviours still occur in clinical environments. This study seeks to explore clinical nurses' experiences and perceptions regarding the factors that lead to discrimination in nursing care.

**Methods:**

This qualitative secondary analysis employed a conventional content analysis. The dataset comprised 13 in‐depth, semi‐structured interviews with clinical nurses working in public and private hospitals in Tehran, Iran. These data were originally collected between June 2023 and May 2024 for two primary studies by the same research team. Purposive sampling with maximum variation was used. The data analysis was conducted manually following the method outlined by Graneheim and Lundman. Trustworthiness was maintained by applying Lincoln and Guba's criteria, including member checking, peer debriefing, and external audit.

**Results:**

Three main categories of factors contributing to discrimination in nursing care were identified: (1) patient‐related factors (including social and economic status, demographic characteristics, clinical condition, and interaction and behavioural characteristics); (2) organizational factors (including hospital policies and guidelines, resource constraints, workload pressures, external influence or interference, and lack of formal education on discrimination and justice); and (3) nurse‐related factors (including receipt of informal benefits and nurses' prior experiences and emotions).

**Conclusion:**

Discrimination in nursing care is a complex, systemic issue that results from the interactions between patient traits, organizational needs, and individual nurse factors. Addressing this effectively involves shifting from a solely individual approach to implementing organizational changes, alleviating systemic pressures, and offering focused education on justice and non‐discrimination within nursing practice.

**Implications for the Profession and/or Patient Care:**

The findings provide a multi‐level causal framework to guide interventions at the point of care, the unit level, and the educational level. Addressing organizational pressures, informal referrals, and educational gaps can reduce discriminatory practices and promote equitable, dignity‐conserving care.

**Impact:**

This study addresses the question of why discriminatory nursing care occurs, moving beyond descriptive accounts to identify actionable contributing factors. The findings benefit nurses, nursing managers, educators, and policymakers by providing an evidence base for targeted interventions.

**Reporting Method:**

The study was reported in accordance with the Consolidated Criteria for Reporting Qualitative Research (COREQ) checklist.

**Patient or Public Contribution:**

No patient or public contribution.

## 1 Introduction

Nursing ethics fundamentally rest on justice and nondiscriminatory care (Karimian et al. 2025). However, evidence shows that discrimination still exists in clinical settings, appearing as structural, systemic inequities and individual biases, often unconscious. This discrimination harms care quality, patient health outcomes, and healthcare trust (Agénor et al. 2022; Tyerman et al. 2020). Multiple lines of evidence indicate that discrimination in clinical settings is a widespread phenomenon. At the international level, a 2023 national survey in the United States found that 60% of Black adults and 42% of Asian adults reported preparing themselves for potentially unfair treatment during medical visits, highlighting the pervasiveness of this issue (KFF 2023).

Nurses, being the largest group of health professionals with continuous patient contact, hold a powerful yet vulnerable role in either sustaining or challenging these inequities (Adynski et al. 2024). The conflict between ethical principles and practical realities has created an urgent moral and practical crisis in nursing.

The Iranian healthcare context adds specific dimensions to this phenomenon. The country faces severe economic sanctions, a depreciating national currency, and chronic shortages of medical supplies and equipment (Mohamadi et al. 2024). The nurse‐to‐patient ratio in Iranian public hospitals (often 1:10‐15 in general wards) far exceeds international standards, contributing to extreme workload, burnout, and high turnover rates (Shamsi and Peyravi 2020). Moreover, the emigration of experienced nurses to other countries has further strained the remaining workforce. These contextual pressures create an environment where discriminatory practices—whether intentional or inadvertent—are more likely to emerge, as nurses are forced to make rapid prioritization decisions with limited resources.

Despite a substantial body of literature on health disparities and inequities in clinical settings, our understanding of the underlying causes of discrimination in nursing practice remains fragmented and incomplete. First, most studies rely on quantitative designs (e.g., surveys measuring prevalence or correlates) and are limited in their ability to explore the “how” and “why” of nurses' discriminatory behaviours (Ayhan et al. 2020). Second, the focus has been on individual‐level factors—such as implicit bias—while the dynamic interplay between these factors and organizational contexts (including ward culture, excessive workload, resource shortages, and ineffective policies) has often been overlooked (Fish and Syed 2019). Third, the geographical and cultural scope of this research is relatively narrow, with most studies conducted in Western settings; findings may not be transferable to non‐Western healthcare systems with different power dynamics, cultural norms, and resource constraints (FitzGerald et al. 2019). Collectively, these gaps pose a significant barrier to the development of effective educational and organizational interventions to reduce discrimination in nursing care (Gutman et al. 2026).

Addressing these gaps is not merely a theoretical concern but a practical necessity with direct implications for patient safety and dignity. Discrimination has been linked to misdiagnosis, delays in care, reduced patient satisfaction, and the exacerbation of health inequalities (Saluja and Bryant 2021). Accordingly, this qualitative study was designed to provide an in‐depth, interpretive exploration of nurses' experiences, perceptions, and ethical tensions in situations that may involve discriminatory care. The central research question guiding this study was:

From nurses' perspectives, what individual, interpersonal, and organizational factors shape behaviours perceived as discriminatory in nursing care?

The present manuscript constitutes a qualitative secondary analysis of data collected for two primary studies conducted by the same research team (Karimian et al. 2025, 2026). The primary studies explored (a) clinical nurses' experiences of discrimination against patients, and (b) nurses' experiences of discriminatory care toward vulnerable patients (older adults and those at the end of life). The present study addresses a distinct research question—the causes and contributing factors of discriminatory nursing care—which was not the focus of either prior publication. The relationship between these reports is detailed in the Methods section.

The contribution of this study is threefold. Methodologically, employing conventional qualitative content analysis captures the deeper cognitive and moral reasoning of nurses that quantitative methods cannot reveal. Theoretically, by examining the interplay of individual (biases), interpersonal (nurse–patient relationships), and organizational factors (workload pressures and prevailing institutional culture), it offers a comprehensive, context‐sensitive framework for understanding the roots of discrimination—an essential foundation for designing effective solutions. Culturally, conducting this study in Iran provides unique and much‐needed insight into how discrimination in nursing care manifests within a distinct sociocultural and health system context.

## 2 Methods

This study is a qualitative secondary analysis that employed a conventional content analysis approach to examine clinical nurses' experiences with patient discrimination. The data were originally collected between June 2023 and May 2024 for two primary studies conducted by the same research team; the present analysis was conducted on this shared dataset. Conventional (inductive) content analysis is particularly suitable when existing theory or research literature on a phenomenon is limited, as it allows codes, categories, and themes to emerge directly from the data without being constrained by a pre‐existing framework (Graneheim and Lundman 2004). This approach enabled participants to articulate their perceptions of discriminatory nursing care, drawing on their experiences and providing specific examples of such practices. The research was conducted in both public and private hospitals in Tehran, Iran, selected for their diverse patient populations with respect to sex, age, culture, and socioeconomic background.

### 2.1 Relationship to Prior Publications

This study is a qualitative secondary analysis of data originally collected for two primary studies by the same research team: the first primary study (Karimian et al. 2025), which explored clinical nurses' experiences of discrimination against patients, and the second primary study (Karimian et al. 2026), which focused on discriminatory care toward older adults and patients at the end of life.

We explicitly confirm that the same 13 participants and the same 13 in‐depth interviews were used across all three publications. All three reports share the same study setting (public and private hospitals in Tehran, Iran), the same data collection period (June 2023 to May 2024), the same interviewer (the first author), the same sampling approach (purposive sampling with maximum variation), and the same saturation process (confirmed after the 11th interview, with two additional interviews for verification). The primary study and data source are therefore identical across all three publications.

However, the three publications are analytically distinct. The 2025 study addressed what forms of discrimination occur; the 2026 study addressed discrimination toward specific vulnerable groups (older adults and end‐of‐life patients); the present manuscript addresses why discrimination occurs—that is, its causes and contributing factors. This shift from descriptive phenomenology to causal attribution constitutes a distinct analytical contribution. The present analysis identified three categories and 10 subcategories (patient‐related, organizational, and nurse‐related factors), none of which overlap with the thematic structures reported in the prior publications. Furthermore, no participant quotations, categories, or subcategories from the present analysis have appeared in either prior publication.

In summary, the overlap between the three reports is limited to the raw dataset only. The research question, analytical framework, categories, subcategories, and all participant quotations in the present manuscript are entirely distinct. This manuscript therefore constitutes a legitimate qualitative secondary analysis rather than a redundant publication, consistent with ICMJE recommendations and established methodological standards for qualitative secondary analysis.

### 2.2 Research Participants

The study included nurses from therapeutic departments directly involved in patient care. Participants were initially selected through purposive sampling based on established inclusion criteria. The recruitment process involved the first author visiting hospital wards, introducing the study, and inviting eligible nurses to participate. Additional participants were recruited using information from the initial group (snowball sampling).

Inclusion criteria were: (a) at least 6 months of active clinical work experience, (b) willingness to participate, and (c) relevant experience related to the study topic. Exclusion criteria were: (a) unwillingness to continue participation, and (b) transfer to a non‐clinical department during the study. The aim was to gather a diverse group of participants reflecting different ages, genders, marital statuses, work experiences, educational backgrounds, departments, and hospital types (maximum variation sampling).

During recruitment, two nurses declined our invitation to participate, both citing heavy workload and lack of time. Recruitment continued until data saturation was achieved, confirmed after the 11th interview, with two additional interviews conducted to verify saturation, yielding a final sample of 13 nurses.

### 2.3 Data Collection

Data were gathered through semi‐structured, face‐to‐face, and online individual interviews. The first author, a licensed nursing PhD student, carried out interviews in Persian from June 2023 to May 2024. He visited hospitals to invite suitable candidates for interviews. During these visits, the purpose and procedures of the study were explained to potential participants. Once agreement was reached on the interview timing and location, data collection commenced. Interviews began with broad questions like, “In your experience, what factors contribute to differences or inequalities in the nursing care provided to patients in clinical settings?” Please share your experience. Follow‐up questions were tailored based on the interviewee's responses. Additional exploratory questions, such as “Could you elaborate further on your statement?” and “Could you provide more context about this specific incident?” were also asked. Interviews took place individually in quiet, private settings and continued until participants felt they had shared all relevant information. During interviews, voices were recorded, and notes were taken. After each interview, participants were encouraged to add more comments, and the possibility of further interviews or follow‐up questions was discussed, along with gratitude for their contributions.

All interviews were conducted in Persian. Verbatim transcripts were translated into English by a bilingual researcher (fluent in both Persian and English, with prior experience in qualitative health research). To ensure conceptual and semantic equivalence, a second bilingual expert independently back‐translated a randomly selected 20% of the translated quotations into Persian. Discrepancies were discussed and resolved through consensus. Particular attention was paid to culturally specific expressions, metaphors, and colloquialisms, which were preserved through explanatory footnotes where literal translation would have obscured meaning.

Data saturation was defined as the point at which further data yield concepts that are conceptually similar to those already identified (Hennink and Kaiser 2022). Using continuous comparative analysis, the researcher compared new data with existing codes and themes to determine if new patterns arose. After the 11th interview, no new codes emerged, and the themes and patterns from previous interviews repeated, with no new insights. To ensure that saturation had genuinely been reached, two additional interviews were conducted; these confirmed that no new information was generated. A review meeting with the research team, including an external qualitative expert, confirmed that data saturation had been achieved.

### 2.4 Researcher Reflexivity and Positionality

The interviewer, a PhD candidate in nursing with clinical experience in Iranian hospitals, conducted all interviews. The interviewer had prior familiarity with the topic of discrimination in nursing care through their clinical background, which facilitated rapport with participants but also risked imposing pre‐existing assumptions. To mitigate this, the interviewer maintained a reflexive journal throughout data collection and analysis, documenting their assumptions, emotional responses, and emerging interpretations. The interviewer had no prior personal or professional relationship with any participant. During interviews, the interviewer deliberately used open‐ended prompts and avoided leading questions. During analysis, two researchers independently reviewed the coding, and an external qualitative expert audited the analytical process. Differences in interpretation were resolved through consensus. The research team included members with diverse methodological and clinical backgrounds, further reducing the influence of any single researcher's perspective.

### 2.5 Data Analysis

The study employed Graneheim and Lundman's content analysis method (2004), which consists of five distinct steps:

1. *Immersion*: Following each interview, the audio recordings were transcribed verbatim, and multiple thorough readings were conducted to gain a deep understanding of the data.
2. *Identification of meaning units*: Significant statements (meaning units) relevant to the research question were identified.
3. *Condensation and labelling*: Meaning units were condensed and labelled with initial codes.
4. *Categorization*: Open codes were extracted and grouped into subcategories and categories based on similarities and differences.
5. *Abstraction*: Categories were reviewed and refined to ensure conceptual coherence

Throughout the research, the team continually reviewed the coding process, with data analysis occurring alongside data collection. An example of this analysis process is shown in Table 1.

**TABLE 1:** An example of the data analysis process.

Direct quote (meaning unit)	Condensed meaning unit	Code	Subcategory	Category
“The head nurse called the ward and stated that this patient is an acquaintance of the hospital director; therefore, provide them with an isolation room to ensure comfort”	Head nurse called to provide an isolation room for an acquaintance of the hospital director	Allocation of best ward room to VIP patient	Patients' socioeconomic status	Patient‐related factors
“If the patient were elderly, we would check vital signs every 12 h rather than every 6 h”	Reduced monitoring frequency for elderly patients	Inadequate monitoring in elderly patients	Patients' demographic characteristics	Patient‐related factors
“We had a patient who was addicted, and none of the nurses would really go to their bedside”	Avoidance of bedside care for addicted patients	Insufficient physical presence for substance users	Clinical condition and type of illness	Patient‐related factors
“The patient insulted the nurses, so we did not administer their antibiotics or IV fluids”	Withholding medications due to patient insult	Withholding prescribed medications from aggressive patients	Interaction and behavioural characteristics	Patient‐related factors

### 2.6 Rigour

This study aimed to ensure reliable and trustworthy outcomes by implementing transparency measures and following Lincoln and Guba's standards (Guba and Lincoln 1994). Several strategies were employed to enhance data credibility:

- *Member checking*: After initial coding and categorization, a summary of findings was shared with four participants, who confirmed that the interpretations accurately reflected their experiences (this was not merely asking follow‐up questions during interviews, but a formal process of verifying interpretations with participants).
- *Peer debriefing*: Two research team members—one with a PhD in nursing and another an expert in qualitative research—independently reviewed the coded data to verify reliability, resolving any disagreements through consensus.
- *External audit*: To support confirmability, an external qualitative expert audited the coding process and the development of categories, verifying that the findings were grounded in the data.
- *Maximum variation sampling*: Sampling was deliberately designed to maximize variance (including different departments, hospital types, ages, genders, and experience levels), thereby improving the transferability of the results.

### 2.7 Ethical Considerations

This study received ethical approval from the Research Ethics Committee of the University of Tarbiat Modares (Ethical Code: IR.MODARES.REC.1402.017). The study's goals, methods, data confidentiality procedures, and voice recordings were explained in detail to the participants, who were also informed that their participation was voluntary. Both written and verbal informed consent were obtained. Participants were assured that their information would be kept confidential and that they could withdraw from the study at any time without repercussions. Audio files and transcripts were password‐protected and accessible only to research team members. All participant names were replaced with pseudonyms (N1–N13).

## 3 Results

### 3.1 Participant Characteristics

The study included 13 clinical nurses. Mean age was 34.7 years, and mean work experience was 10.8 years (calculated from participants' exact reported values, including months). Due to distance, interviews with three nurses were conducted online via video call. All demographic data were verified against original study records (signed consent forms, interview logs, and the principal investigator's master data sheet) prior to submission. Table 2 presents the participants' profiles.

**TABLE 2:** Demographic characteristics of the participants.

Alias	Gender	Age (years)	Marital status	Educational level	Work experience (years)	Department	Hospital setting	Interview duration (minutes)
N1	M	38	Married	PhD	16	Psychiatric and burns	State (educational)^a^	44
N2	F	52	Single	Bachelor	28	Surgery	Private	37
N3	F	44	Married	Bachelor	12	ICU	Private	27
N4	F	35	Single	Bachelor	11	Neurosurgery	State (non‐educational)^b^	41
N5	M	27	Single	Bachelor	4	Emergency	Private	36
N6	M	35	Married	Master	10	Emergency	State (educational)	41
N7	M	27	Single	Bachelor	4	Internal medicine	Private	28
N8	M	30	Single	Bachelor	8	Emergency	State (non‐educational)	30
N9	F	50	Married	Master	19	Surgery and orthopaedics	Private	42
N10	M	28	Single	Bachelor	3	ICU	Private	35
N11	F	35	Married	Bachelor	11	Infectious	State (educational)	26
N12	F	30	Married	Bachelor	7	Internal and emergency procedures	State (educational)	25
N13	F	24	Single	Bachelor	2	NICU	State (educational)	35

*Note:* Mean age (34.7 years) and mean work experience (10.8 years) were calculated from participants' exact reported values, including months (e.g., “10 years and 4 months”). Table values are rounded to whole years for readability. All individual values were verified against original study records.

Abbreviations: F, female; ICU, intensive care unit; M, male; NICU, neonatal intensive care unit.

^a^ State (educational): Public hospitals affiliated with medical universities, serving both clinical care and teaching purposes.

^b^ State (non‐educational): Public hospitals not formally affiliated with a medical university, primarily providing clinical services.

### 3.2 Qualitative Findings

Through qualitative content analysis of in‐depth interviews with 13 clinical nurses, three main categories of factors contributing to patient discrimination were identified: patient‐related, organizational, and nurse‐related factors. Each category comprised several subcategories, presented and elaborated below (Table 3).

**TABLE 3:** Categories and subcategories derived from qualitative content analysis.

Category	Subcategory
Patient‐related factors	Patients' socioeconomic status
	Patients' demographic characteristics
	Clinical condition and type of illness
	Interaction and behavioural characteristics
Organizational factors	Internal hospital policies and regulations
	Operational conditions and organizational constraints
	Influence of actors external to the hospital
	Lack of formal education on discrimination and justice in care
Nurse‐related factors	Informal benefits, relationships, and rewards
	Nurses' prior experiences and emotional responses

### 3.3 Category 1: Patient‐Related Factors

#### 3.3.1 Subcategory 1.1: Patients' Socioeconomic Status

The findings revealed that patients' socioeconomic status was among the most prominent determinants of discriminatory practices in nursing care. Nurses frequently reported that patients with greater economic capital, social visibility, or power‐related connections—such as wealthy individuals, celebrities, or government officials—received more attention, faster therapeutic interventions, and preferential access to facilities. These differences were evident not only in formal clinical decisions but also in routine caregiving behaviours. One participant stated:

Patients who are wealthy or well‐known are treated completely differently. Everyone pays more attention to them; their medications are administered sooner, the physician arrives more quickly, and they are usually assigned the best room in the ward. They are prioritized in almost everything. (N13)

In contrast, many interviews highlighted neglect, reduced motivation, or minimal care for patients of lower social status or those carrying social stigma. Nurses particularly referred to prisoners, individuals with substance use disorders, homeless patients, and those lacking social support as groups receiving less attention. One participant explained:

When a prisoner or an addicted patient is admitted, an unconscious distance is created. Care is often provided merely to fulfill the duty, nothing more. We visit them less frequently, communicate less, and overall, the level of care is lower. (N8)

#### 3.3.2 Subcategory 1.2: Patients' Demographic Characteristics

Interview findings indicated that patients' age and gender influenced the prioritization of care and the quality of nursing care. Nurses reported that in daily clinical decision‐making, younger patients generally received more attention, explanation, and follow‐up than older patients. This tendency was particularly pronounced among young female patients and was often justified by beliefs such as “they have a better chance of recovery” or “they should not develop complications.” One participant noted:

When a young patient comes in, especially if she is a woman, we unconsciously become more sensitive; we explain more, monitor vital signs more carefully, and check on her more often. (N3)

In contrast, a different pattern of care was observed among older adults. Although elderly patients were monitored more frequently due to frailty, comorbidities, and greater need for basic care, some nurses acknowledged that therapeutic expectations for this group were lower and that the intensity of treatment follow‐up was sometimes reduced. One nurse explained:

We monitor older patients more—blood pressure, blood sugar, respiratory status—but honestly, sometimes we don't pursue aggressive treatment as much because we say they are old and have underlying diseases. (N7)

Additionally, participants noted that gender‐related stereotypes influenced nursing behaviour. Female patients were often perceived as more vulnerable and in need of emotional support and detailed explanations, whereas male patients were implicitly expected to tolerate discomfort or to complain less. Overall, this subcategory indicates that age and gender affect not only clinical assessment but also empathy, communication style, and the intensity of nursing follow‐up.

#### 3.3.3 Subcategory 1.3: Clinical Condition and Type of Illness

The findings showed that patients in end‐stage or terminal phases of illness often experienced a gradual decline in the intensity, depth, and continuity of nursing care. Nurses reported that feelings of “futility of treatment,” “proximity to death,” and “lack of hope for recovery” could unconsciously lead to emotional distancing and reduced caregiving attention. One participant stated:

When you know the patient is end‐stage and there's really nothing more you can do, your focus unconsciously shifts toward patients who still have a chance of recovery. (N1)

Some nurses acknowledged that, under such circumstances, interventions such as pain control, suctioning, continuous monitoring, or prompt responses to patient requests might be reduced, and care would become largely routine and minimal. As one nurse described:

For end‐stage patients, care is sometimes provided just to keep them alive, not to make them comfortable. (N5)

Furthermore, the findings indicated that the infectious nature of specific diseases—particularly those associated with social stigma, such as HIV/AIDS, hepatitis, and HPV—served as an independent factor that reduced both the quality and quantity of nursing care. Nurses, implicitly or explicitly, cited fear of contamination, concerns about disease transmission, and negative stereotypes. One participant noted:

When ‘HIV positive’ or ‘hepatitis’ is noted in the chart, some colleagues unconsciously keep their distance; even tasks that should be done quickly are delayed. (N11)

In some cases, these patients were subjected to excessive caution, avoidance of direct contact, and reduced human interaction—behaviours that went beyond standard universal precautions.

#### 3.3.4 Subcategory 1.4: Interaction and Behavioural Characteristics

Patients' behaviours and those of their family members also influenced the delivery of care. Nurses reported that calm, cooperative patients generally received better care, whereas aggressive or demanding patients often received less time and attention. One participant explained:

Ill‐tempered and aggressive patients make the nurse's job harder; we consciously or unconsciously try not to get too involved with them. But if a patient's family member is proactive and constantly follows up, we feel more obligated to report and to provide more care. (N9)

Another nurse added:

When a patient is constantly ringing the bell or making excessive demands, we unconsciously start delaying our response. We know it's not right, but it happens. (N4)

### 3.4 Category 2: Organizational Factors

#### 3.4.1 Subcategory 2.1: Internal Hospital Policies and Regulations

Interview results showed that certain discriminatory care practices were not just due to personal nursing choices but were directly influenced by hospital policies, expectations, and internal rules. Nurses—especially in private hospitals—stated that management, whether formally or informally, expected them to deliver enhanced care and attention to patients classified as ‘special’ or socially influential.

Several nurses reported receiving direct calls or reminders from senior hospital managers emphasizing the importance of maintaining the hospital's reputation, patient satisfaction, and institutional branding. One participant commented:

Many times, the hospital director or manager calls and says this patient is important and to take extra care of them. They clearly say this patient affects the hospital's reputation and brand. (N12)

Nurses reported that in such situations, even when the patient's clinical condition was no different from others', they were instructed to provide care more carefully, rapidly, and sensitively. As one nurse explained:

These patients aren't necessarily sicker, but there's an emphasis on doing everything for them without delay—from medications to physician visits. (N4)

Although such directives are often justified by the competitive logic of private hospitals and patient satisfaction goals, they create structural inequalities in the allocation of nursing attention and resources. As one participant noted:

When orders come from above to provide more care, our professional autonomy is taken away—even if we know other patients need it just as much. (N6)

Overall, this subcategory demonstrates that discrimination in healthcare can stem from institutional policies and managerial decisions. In these situations, nurses are less the cause of discrimination and more the enforcers of organizational directives that compromise fairness in care.

#### 3.4.2 Subcategory 2.2: Operational Conditions and Organizational Constraints

The findings revealed that shortages of both human and material resources significantly contributed to unintentional discrimination in nursing care. Nurses consistently mentioned staff shortages, insufficient medical equipment, limited bed capacity, and high workloads—factors that compelled them to prioritize patients and make quick, sometimes unfair decisions.

When there's both a staff shortage and a bed shortage, you just think about which patient you can manage faster—not who deserves care more. (N11)

Some nurses observed that, in these conditions, patients with simpler treatment plans, higher cooperation, or easier service access were attended to sooner. Meanwhile, complex, high‐risk, or more time‐consuming patients were unintentionally deprioritized. One participant noted:

When you know that caring for one patient will take a lot of time and energy, you unconsciously go to the bottom of the list—even if you know it's ethically wrong. (N7)

Nurses highlighted that this type of discrimination was not due to personal intent but resulted from systemic pressures, burnout, and structural limitations. Overall, this category shows that challenging operational conditions can force nurses to stray from their ideal caregiving role and lead them to make urgent decisions, which can perpetuate disparities in access to care.

#### 3.4.3 Subcategory 2.3: Influence of Actors External to the Hospital

Findings indicated that discrimination in nursing services often stemmed from pressure exerted by influential figures and unofficial referrals outside formal care structures. Nurses commonly reported experiencing direct or indirect pressure from senior physicians, hospital administrators, supervisors, or known contacts within the healthcare system—pressures that influenced care decisions and resulted in preferential treatment for certain patients. Participants noted that these referrals were usually communicated via phone calls, informal messages, or verbal reminders, often with phrases like “keep an eye on this patient” or “please take extra care.” One nurse remarked:

Often a call comes saying the patient is an acquaintance or that the doctor has emphasized it. That one sentence is enough for everyone to know this patient should be treated differently. (N3)

Consequently, measures like quicker medication administration, increased bedside visits, faster physician coordination, or earlier referrals to diagnostic and ancillary services were put into practice—even when the patient's clinical condition was not significantly different from others. One participant remarked:

Sometimes the referred patient isn't clinically urgent at all, but because they're recommended, their care takes precedence over everyone else's. (N11)

Some nurses highlighted that neglecting these referrals might lead to professional or organizational issues, such as managerial warnings, less support, or conflicts with senior doctors. One participant noted:

If you ignore a recommendation, you might face problems later in your shift or during your performance evaluation. Often, you have no choice but to comply. (N4)

Overall, this subcategory demonstrates that external influence acts as an informal but strong factor that shifts care from clinical needs to priorities based on power, thus perpetuating discrimination through everyday, seemingly minor choices.

#### 3.4.4 Subcategory 2.4: Lack of Formal Education on Discrimination and Justice in Care

Interview findings revealed that a lack of formal, structured education on discrimination, justice in care, and ethical sensitivity significantly contributed to discriminatory behaviours. Most nurses stated that their academic or in‐service training rarely included specific instruction on identifying discrimination, understanding its effects, or preventing it. Although topics like nursing ethics or patients' rights were discussed generally, actual instances of discrimination in clinical practice were seldom addressed. One participant noted:

No one ever told us that what we're doing could be considered discrimination. They just said ‘observe professional ethics,’ but they never explained it with real examples. (N2)

Without explicit education, discriminatory behaviours tend to develop unconsciously, influenced by workplace norms. Many nurses admitted to copying the behaviour of more experienced staff or following the ward's established culture. Participants highlighted that proper training could have heightened their awareness and prompted alternative choices. In the absence of such education, discrimination was often seen as a normal part of the system, viewed as an unavoidable result of workload stresses or systemic healthcare challenges.

Another nurse elaborated:

We have in‐service education on CPR, infection control, and new equipment, but never on how to treat all patients equally. It's as if they assume we already know, but we don't—not in practice. (N8)

### 3.5 Category 3: Nurse‐Related Factors

#### 3.5.1 Subcategory 3.1: Informal Benefits, Relationships, and Rewards

Nurses recognized that tips, gifts, or implicit promises from patients or their families could inadvertently affect how they prioritize care. These rewards usually occurred outside official organizational policies and often manifested in subtle yet meaningful ways. Some participants noted that even without conscious intent to discriminate, receiving material or emotional benefits fostered a sense of reciprocal obligation. This sometimes led to faster care, closer follow‐up, or spending extra time with specific patients. One nurse remarked:

When a patient or family does you a favor, you unconsciously feel you should compensate—even if you know professionally that it's not right. (N1)

In addition to direct advice, nurses mentioned informal networks and favouritism, such as personal contacts, hometown ties, referrals from influential people, and family links. One participant noted:

When you realize a patient is an acquaintance or someone important has introduced them, a special sensitivity develops—you don't want your name associated with a negative outcome. (N12)

In certain instances, these informal relationships came with implicit expectations from patients or families, creating ethical dilemmas for nurses. Participants observed that denying preferential treatment might result in dissatisfaction, complaints, or strained professional relationships.

#### 3.5.2 Subcategory 3.2: Nurses' Prior Experiences and Emotional Responses

Finally, nurses' personal experiences and emotional responses influenced caregiving practices. Some participants reported greater empathy when patients' situations resembled their own or those of their family members. For example, one nurse stated:

When I see a young patient or a child whose condition resembles my own child, I do everything I can and feel like I'm caring for my own child. (N13)

Conversely, others noted that caring for high‐risk or terminal patients sometimes evoked feelings of futility, which could reduce the intensity of care. As one nurse explained:

Sometimes when I see a patient in the final stage of cancer and recovery is impossible, I unconsciously follow up less; I feel that whatever I do won't make a difference. (N6)

Another nurse shared:

I had a patient who reminded me of my father who passed away. I found myself spending extra time with him, talking to him more, and checking on him more often than other patients. I realised later that this wasn't fair to others. (N9)

These findings indicate that nurses' emotional burden and personal empathy can both enhance and diminish caregiving efforts, depending on the clinical context.

## 4 Discussion

The findings of this qualitative study indicate that discrimination in the provision of nursing care is a multidimensional and complex phenomenon arising from the interplay among patient‐related characteristics, organizational structures, and nurse‐related factors. A content analysis of interviews with clinical nurses revealed that discrimination is not merely the result of individual attitudes; rather, it is shaped simultaneously by power relations, managerial logics within the healthcare system, structural constraints, and educational gaps.

The results indicate that patients with higher social and economic capital—such as wealthy individuals, celebrities, or those with political or professional influence—consistently receive preferential treatment. Conversely, socially stigmatized patients (e.g., prisoners, individuals with substance use disorders, or homeless persons) often get minimal care. These findings highlight how social inequality can directly influence healthcare access, as healthcare professionals—often unconsciously—reproduce broader social hierarchies. This pattern aligns with previous research showing that nurses in clinical settings face implicit pressures to align their care practices with patients' social standing. Such dynamics can undermine principles of justice, human dignity, and needs‐based care (Nutbeam and Lloyd 2021; Hosseinabadi‐Farahani et al. 2023).

This study highlighted the significant impact of age and gender on nursing care. It was common to prioritize younger patients, especially young women, often based on beliefs of a “higher likelihood of recovery” or greater value placed on their future. In contrast, older adults tended to receive less intensive monitoring and follow‐up as their treatment expectations decreased. These patterns support the idea of ageism in healthcare, where older patients may be implicitly seen as less deserving of aggressive treatment (Fernández‐Puerta et al. 2024; Chang et al. 2020). As Fernández‐Puerta et al. (2024) noted, ageism among healthcare workers is often subtle and unconscious, manifesting as reduced communication, less shared decision‐making, and lower expectations for recovery. Our study extends these findings by showing how ageist attitudes intersect with organizational constraints (e.g., high workload) to produce tangible disparities in care intensity and follow‐up. This is particularly concerning given Iran's rapidly aging population and the increasing demand for geriatric nursing expertise (Karimian et al. 2026). Additionally, gender stereotypes influence nurse–patient interactions, reflecting enduring cultural biases that can result in unequal care experiences (Cuevas et al. 2025).

A significant finding was that patients in end‐stage conditions and those with stigmatized infectious diseases like HIV, hepatitis, and HPV received lower‐quality nursing care. Nurses often reported less physical presence, limited communication, and a shift to minimal, task‐focused care for these groups. These behaviours were frequently linked to feelings of treatment futility, reduced hope for positive outcomes, fears of infection spread, and stigma related to these conditions. These results align with existing research indicating that patients at the end of life and those with stigmatized illnesses are more prone to depersonalized care, less empathy, and unequal clinical attention (Feyissa et al. 2019; Ferguson et al. 2022). Such practices conflict with fundamental principles of palliative care, dignity‐centred care, healthcare justice, and professional nursing ethics, potentially significantly harming patients' care experiences (Maffoni et al. 2019).

The study also revealed that discriminatory care often stems from organizational policies, especially in private hospitals where competition, patient satisfaction, and branding are emphasized. Managerial directives to give special attention to “VIP” or referred patients show how institutional and structural discrimination is maintained (Hastings and Domegan 2013). In these settings, nurses act not as independent agents of discrimination but as enforcers of organizational rules. This underscores the idea of moral distress, where nurses know the right ethical action but are unable to act due to constraints (Lamoureux et al. 2024).

This study also showed that factors related to nurses—like receiving informal tips, personal connections, and previous experiences—can affect how they provide care. Furthermore, the lack of formal education specifically on justice and discrimination in care makes these behaviours seem normal, so nurses may not realize their ethical significance. This aligns with earlier research suggesting that insufficient education, informal learning at work, and a lack of ethical support help perpetuate discriminatory practices in clinical settings (Karimian et al. 2025; Hosseinabadi‐Farahani et al. 2023).

Discrimination in nursing care is not always driven by malicious intent; it can stem from incomplete professional socialization and implicit habits learned unconsciously. Nurses may unknowingly adopt unfair practices rooted in workplace culture (Johnstone 2022). Therefore, nursing education should incorporate practical training that focuses on justice, recognizing discrimination, and ethical decision‐making within real clinical scenarios to enhance nurses' moral awareness and skills.

This study examined nurses' experiences of discrimination in Iran's nursing care and highlighted aspects often missed in quantitative research. As with any study, its limitations must be recognized.

The present study constitutes a qualitative secondary analysis of data reported in two prior publications by the same research team (Karimian et al. 2025, 2026). While the prior studies described the manifestations and experiences of discrimination, the present analysis provides a distinct contribution by mapping the causes and contributing factors of discriminatory care across three levels (patient‐related, organizational, nurse‐related). This causal framework is directly actionable for intervention design and complements, rather than duplicates, the descriptive findings of the primary studies.

### 4.1 Limitations

Several limitations should be acknowledged. First, social desirability bias may have influenced participants' responses; given the sensitive nature of discrimination, some nurses may have under‐reported their own discriminatory behaviours or those of their colleagues. Second, the study was conducted in Tehran, Iran, and findings may not be directly transferable to other regions with different cultural, economic, or healthcare contexts. Third, despite our efforts to protect anonymity, the detailed demographic information provided in Table 2 might, in a small professional community, allow for the identification of certain participants. We mitigated this by aggregating some demographic variables and emphasizing confidentiality in all communications. Fourth, our sample comprised nurses from urban public and private hospitals; the perspectives of nurses working in rural areas, primary care, or home health settings remain unexplored. Fifth, the cross‐sectional nature of qualitative interviews captures experiences at a single point in time; longitudinal studies could reveal how discriminatory behaviours evolve.

### 4.2 Implications for Nursing Practice

The findings of this study have direct implications for nursing practice at three levels:

- *At the point of care*: Nurses should engage in structured reflective practice to identify unconscious prioritization patterns. Simple strategies—such as routine self‐auditing of time spent per patient and standardizing communication protocols across all patient groups—can reduce inadvertent disparities.
- *At the unit and organizational level*: Nursing managers should implement transparent triage and prioritization protocols based on clinical urgency, establish anonymous reporting systems for discriminatory practices, and provide regular ethics rounds where moral distress can be discussed without fear of reprisal.
- *At the educational level*: Undergraduate and continuing education curricula should include specific modules on recognizing implicit bias, the social determinants of health, and the ethical foundations of non‐discriminatory care—moving beyond abstract principles to case‐based learning.

These recommendations are grounded in the specific factors identified by participants (workload pressures, informal referrals, lack of formal education) rather than generic prescriptions.

## 5 Conclusion

This study reveals that discrimination in nursing care is a complex, multi‐layered issue influenced by patient attributes, organizational frameworks, and individual nurse factors. Often, such discrimination remains unconscious, embedded in structural and institutional practices, rather than just stemming from nurses' personal beliefs or intentions. The findings indicate that addressing discrimination requires more than just focusing on nurses' ethics. It is crucial to revise organizational policies, lessen structural pressures, clarify clinical guidelines, and include targeted education on justice, social stigma, and discrimination within nursing programs. Additionally, fostering supportive environments where ethical conflicts can be openly discussed and moral distress reduced can significantly help promote fair and equitable care.

This study adds to the existing literature in three significant ways: (1) it is one of the first in‐depth qualitative investigations of discrimination in nursing care within the Middle Eastern context, thereby addressing a substantial geographical gap in the evidence base; (2) it provides a comprehensive, multi‐level framework—encompassing patient, organizational, and nurse‐related factors—that moves beyond individual‐level explanations and captures the systemic nature of the phenomenon; and (3) it offers actionable, context‐sensitive recommendations for policymakers, nursing managers, and educators in resource‐constrained settings, derived directly from the lived experiences of clinical nurses. Ultimately, this study highlights the importance of shifting from an individual‐focused to a system‐focused approach when studying discrimination in nursing care and designing interventions, emphasizing that sustainable change requires addressing structural inequities alongside individual attitudes.

## Author Contributions

**Aram Karimian:** conceptualization, investigation, validation. **Afsaneh Sadooghiasl:** conceptualization, investigation, methodology, validation, project administration. **Mitra Khoobi:** writing – original draft, visualization, data curation. **Eesa Mohammadi:** investigation, writing – original draft, validation. **Anoshirvan Kazemnejad:** validation, formal analysis, data curation.

## Funding

The authors have nothing to report.

## Disclosure

No generative AI was used in the preparation of this manuscript.

## Ethics Statement

The study was approved by the Ethics Committee at Tarbiat Modares University, with the ethics code IR.MODARES.REC.1402.017.

## Conflicts of Interest

The authors declare no conflicts of interest.

## Data Availability

The data that support the findings of this study are available from the corresponding author upon reasonable request.
